# Analysis of anthropometric outcomes in Indian children during the COVID-19 pandemic using National Family Health Survey data

**DOI:** 10.1038/s43856-024-00543-6

**Published:** 2024-07-01

**Authors:** Amit Summan, Arindam Nandi, Ramanan Laxminarayan

**Affiliations:** 1 One Health Trust, 5636 Connecticut Avenue NW, PO Box 42735, Washington, DC 20015 USA; 2 The Population Council, 1 Dag Hammarskjold Plaza, New York, NY 10017 USA; 3 One Health Trust, Obeya Pulse, First Floor, 7/1, Halasur Road, Bengaluru, Karnataka 560042 India; 4 High Meadows Environmental Institute, Princeton University, Guyot Hall, Princeton, NJ 08544 USA

**Keywords:** Viral infection, Public health, Epidemiology, Paediatric research

## Abstract

**Background:**

Disruptions in food, health, and economic systems during the COVID-19 pandemic may have adversely affected child health. There is currently limited research on the potential effects of the COVID-19 pandemic on stunting, wasting, and underweight status of young children.

**Methods:**

We examine the short-term associations between the pandemic and anthropometric outcomes of under-5 children (*n* = 232,920) in India, using data from the National Family Health Survey (2019–2021). Children surveyed after March 2020 are considered as the post-COVID group, while those surveyed earlier are considered as pre-COVID. Potential biases arising from differences in socioeconomic characteristics of the two groups are mitigated using propensity score matching methods.

**Results:**

Post-COVID children surveyed in 2020 and 2021 have 1.2% higher underweight rates, 1.2% lower wasting rates, 0.1 lower height-for-age z-scores (HAZ), and 0.04 lower weight-for-height z-scores as compared with matched pre-COVID children. Post-COVID children surveyed in 2020 have 1.6%, 4.6%, and 2.4% higher stunting, underweight, and wasting rates, respectively, and 0.07 lower HAZ, as compared with matched pre-COVID children. Reductions in nutritional status are largest among children from households in the poorest wealth quintiles.

**Conclusions:**

These findings indicate a trend towards a recovery in child anthropometric outcomes in 2021 after the initial post-pandemic reductions. The resilience of health and food systems to shocks such as COVID-19 should be strengthened while immediate investments are required to decrease child malnutrition and improve broader child health outcomes.

## Plain language summary

This study examined how the COVID-19 pandemic affected the health of children under five years of age in India. We compared children surveyed before and after the pandemic. We find that children surveyed after the pandemic began in 2020 had decreased height and weight when compared to pre-pandemic measurements. In 2021, these outcomes improved but some outcomes, primarily weight, did not recover completely. These effects were most pronounced in the poorest households. Overall, our findings suggest that some of the effects of the pandemic may be short-term, but these require further study. Investments are required to reduce child malnutrition and improve the resilience of health and foods systems to shocks.

## Abstract (web summary)

Summan et al. estimate changes in anthropometric outcomes in Indian children during the COVID-19 pandemic. They find short-term increased adverse growth events such as stunting and lower weight with trends towards recovery in 2021.

## Introduction

The COVID-19 pandemic posed unprecedented challenges to health and economic systems globally. Governments around the world responded with non-pharmaceutical interventions (NPIs) such as lockdowns and travel restrictions – especially before widespread vaccine availability—which limited mobility and caused economic shocks^1–3^. Health systems were overwhelmed and resources were diverted from routine to COVID-19-related care^4^. These breakdowns potentially affected health in excess of the direct effects due to COVID-19 infection—as of 2021, 15 million excess deaths globally have been attributed to the combined direct and indirect effects of COVID-19^5^, of which 3–4 million deaths were estimated to be in India^5–9^. Newer data at the regional level support these excess mortality estimates. For example, surveillance data from Madurai, a large city in India, shows that all-cause deaths were 30% higher than expected levels between March, 2020 and July, 2021^10^.

Beyond pediatric COVID-19 infections, the pandemic in India may have affected child health in additional ways. India entered a complete national lockdown on March 25, 2020, which suspended public transit and prohibited all gatherings, and only allowed essential services to operate. After the lockdown was lifted on June 1, 2020, local containment measures and other NPI restrictions continued for several months. During this time, resources were diverted from maternal and child healthcare programs to pandemic-related care, which may have adversely affected birth outcomes and early childhood health^11,12^. A modeling study estimated that during the six months starting in May 2020, reduced access to antenatal and postnatal care, immunization, and preventative child healthcare due to the pandemic could have resulted in 253,500 additional child deaths and 12,200 additional maternal deaths across 118 low- and middle-income countries (LMICs)^13^. In India, coverage and timely receipt rates of routine childhood vaccines were estimated to reduce by 2–10% due to the pandemic^14^. There were also large reductions in antenatal care seeking, emergency obstetric care delivery, and institutional childbirth rates because of the pandemic in India^11^.

Food system disruptions may have also affected child health and nutrition. Global food production and delivery systems operated at limited capacity due to worker shortages and supply chain bottlenecks^15^. In 2020, the number of food-insecure people rose by 28% (211 million) globally^16^. In India, wheat prices increased by 4% and rice prices increased by 11% from March 2020 to May 2020, and the national economy contracted by 6.6% through the end of 2020^17,18^. A lack of food security may have impacted child nutrition. Modeling estimates projected that pandemic-induced disruptions in economic, food, and health systems could have resulted in an additional 9.3 million low weight-for-height (wasting) and 2.6 million low height-for-age (stunted) children by 2022 in LMICs^15^. Stunting, wasting, and underweight status are associated with higher levels of morbidity and mortality in childhood^15,19,20^. These effects may represent a lower bound of the negative consequences since poor nutrition during the first 1000 days of life could have a lasting impact on health, schooling, and economic outcomes into later childhood and adulthood^21,22^.

There is limited research on the potential effects of the COVID-19 pandemic on stunting, wasting, and underweight status of young children. Predictive modeling studies^13,15,23,24^ based on projections of the early trajectory of the pandemic may not accurately reflect the true impact of the pandemic on child health due to the inherent uncertainties of such analysis. Empirical investigation of the impact remains limited due to a lack of data. A yet unpublished study using national data^25^ estimated that children born during the COVID-19 pandemic in India weighed 8.98 g less than children born before the pandemic. Another study based on data from a single health center in Mumbai found that the rate of preterm birth—babies born alive before 37 weeks of pregnancy are completed—decreased from 14% to 10% from the first wave to the second wave of the pandemic, although the authors did not control for confounding factors^26^. Preterm birth is a known contributing factor for stunting, wasting, and underweight status in infants^27^.

In this study, we provide the first national estimates of the associations of the COVID-19 pandemic with anthropometric outcomes of children under the age of five years in India during late 2020 and early 2021. Even before the pandemic, India had among the highest prevalence of childhood undernutrition globally. An estimated 36% of India’s 120 million under-5 children were underweight in 2016^28^. Considering that underweight prevalence has improved slowly over recent years, from 43% during 1998–1999, the COVID-19 pandemic could potentially roll back progress by several years^28^.

We used data from the fifth round of the National Family Health Survey, 2019–2021 (NFHS-5) of India. We considered under-5 children who were surveyed after March 25, 2020 (the first date of national lockdown) as the post-COVID group, i.e., those who experienced systemic shocks such as food insecurity, reduced access to healthcare, lower immunization rates, and economic instability due to the pandemic. In comparison, under-5 children surveyed prior to March 25, 2020, were considered as the pre-COVID group. To our knowledge, these are the first national estimates of the associations of the COVID-19 pandemic with child nutritional status in India or any large low- and middle-income country. Our findings indicate that Indian children measured after the pandemic had higher stunting, underweight, and wasting rates, and lower height-for-age z-scores as compared with similar children who were measured before the pandemic.

## Methods

### Data and outcome variables

We used data from the fifth round of the National Family Health Survey (NFHS-5)^29^, a cross-sectional, nationally representative demographic and health survey in India conducted by the International Institute for Population Sciences, which is supported by the Ministry of Health Family Welfare. Phase 1 of the survey was conducted from June 2019 to January 2020, covering 22 states and union territories (UTs) and phase 2 was conducted from January 2020 to April 2021, covering the remaining 14 states and 3 UTs (Supplementary Table 7). Due to COVID-19 lockdowns, survey activities ceased in April 2020 and resumed in November 2020. The survey collected data from 232,920 children under the age of five years (those born since 2016) in 636,699 households across 707 districts of India.

We examined the following growth outcomes of under-5 children: stunting, wasting, underweight, height for age z-scores, and weight for age z-scores (WHZ). Z-scores were calculated based on WHO Child Growth Standards. While wasting or underweight status can reflect both recent acute weight loss or a measure of cumulative malnutrition from birth, stunting is considered to be a function of cumulative infections and nutrition since birth or even from the in-utero stage. Stunting, wasting, and underweight status, were binary variables with a value of 1 if the child was more than two standard deviations lower in height-for-age, weight-for-height, and weight-for-age from the WHO reference median, respectively.

We considered the effects of the COVID-19 pandemic to begin on March 25, 2020, which was the first date of the national lockdown in India. We considered children surveyed by NFHS-5 after the start of the pandemic (those surveyed from November 2020 to April 2021) to be in the post-COVID group. Children surveyed before the pandemic (June 2019–March 2020) were included in the pre-COVID group. These definitions were based on the timing of data collection and not based on whether a child was infected with COVID-19 or exposed to COVID-19 (close contact with an infected person) as NFHS-5 did not collect data on infections or exposure. Our analysis therefore captured the broad population-level effect of the pandemic and related shocks to the health, food supply, and economic systems on child health.

We used publicly available anonymized data from NFHS-5 survey that received ethics clearance from the International Institute for Population Sciences of India. No separate ethics clearance was necessary for this study due to the anonymized nature and public availability of the data.

### Propensity score matching analysis

We used propensity score matching (PSM) to estimate the associations of COVID-19 with child health outcomes. PSM is a quasi-experimental approach used to analyze the effects of interventions in non-experimental data^30,31^. In observational data, background characteristics such as socioeconomic or demographic factors often differ systematically between the intervention and control groups. If these differences are also correlated with the outcome indicator, a comparison of unadjusted group means or ordinary least square estimates of the association between the intervention status and outcome will be biased. Children in the post-COVID group were solely from NFHS-5 phase 2 states as compared with the pre-COVID group that had children from both phase 1 and phase 2 states. If inherent differences between the two groups (e.g., standard of living) are not adequately accounted for, they could influence perceived differences in child growth outcomes. For example, if phase 2 of the survey consists of richer states or those with better health systems on average, least squares estimates of the negative association between the pandemic and child growth outcomes may be smaller in magnitude than the true parameter.

PSM reduces the differences in observed characteristics of the two groups. It matches each post-COVID child with a child who was pre-COVID but had a similar probability of ‘being post-COVID’ based on observable characteristics. After matching, the difference in outcomes between post-COVID and pre-COVID children would be attributable to the pandemic assuming that unobservable factors were evenly distributed between the two groups. The average difference in outcomes between the two matched groups is known as the average treatment effect on the treated (ATT)^30–33^.

We employed a probit model to regress the binary indicator of whether a child was in the pos-COVID group on a set of covariates which included indicators of the state of residence, type of residence (urban vs. rural), wealth index quintile, religion, caste, household size, sex of household head, marital status of the mother, mother and household head’s education level and age, mother’s height, child’s age in months, sex, birth order (first, second, third, fourth or higher), and a binary indicator of whether the child was born in a health facility (instead of home birth). Wealth index was a composite index of household ownership of durable assets such as TV, radio, and car, along with housing condition indicators such the type of construction material, and the availability of toilet and electricity^29^. Meta-analyses have indicated that these variables are all associated with the nutritional status of children^34–36^. Social and economic status, sex, age, and education level of the household head, and place of residence of child’s household may be associated with access to resources and nutritious foods. Child sex, birth order, and household size may affect intrahousehold resource allocation for the child relative to others within the household. Mother’s education level and place of delivery may reflect the quality of parenting and level of investment in child health. Mother’s height impacts child birth outcomes—for example, mothers with short stature are more likely to have babies with low birth weight and small for gestational age status^37^. These children may also experience lower than average physical growth rates.

Using the predicted probability (known as the propensity score) from this regression, we matched each post-COVID child with a pre-COVID child. We used one-to-one, nearest-neighbor matching with replacement. Heteroskedastic-consistent analytical standard errors were used^38^. After matching, we examined the average difference in child growth outcomes across all matched pairs of post-COVID and pre-COVID children. These estimators can be interpreted as the ATT effect of the pandemic.

### Sensitivity analysis and matching quality tests

In a sensitivity test, we accounted for possible differences in past trends in nutritional status between the pre- and post-COVID groups. We repeated our analysis after including three indicators of past nutrition as covariates in our propensity score matching analysis: percentage of under-5 children that were 1) stunted, 2) wasted, and 3) underweight. These indicators were obtained, at the state level, from the National Family Health Survey 2015-2016 (NFHS-4) and combined with the child-level NFHS-5 data in our analysis. Minor differences in state boundaries between the two survey rounds were adjusted in the following way. Jammu and Kashmir (J&K) estimates from NFHS-4 were assigned to J&K and Ladakh in NFHS-5, and estimates of Daman & Diu and Dadra & Nagar Haveli were combined due to small sample sizes.

In further sensitivity analysis, we also examined other matching algorithms—matching of observations to the nearest three neighbors and kernel matching. In kernel matching, each treated observation is matched with a weighted average of control observations, where the weight is an inverse function of the distance between control and treatment observation propensity scores. We imposed “common support” in all models—all observations below the minimum or above the maximum propensity score for the post-COVID group were excluded.

Previous analysis has shown that health service delivery improved partially in late 2020^14^, which could have potentially improved child anthropometric outcomes in 2021. To capture such trends, we separately analyzed the entire sample of post-COVID children (surveyed in 2020 and 2021) and those surveyed in 2020. The comparison group for both analyses were the same (pre-COVID children surveyed prior to the first lockdown). It allowed us to understand the potential medium-term and short-term effects of the pandemic. We also conducted subsample analysis, restricting the sample to male or female children, and rural, urban, high-wealth (top three wealth quintiles), and low-wealth (bottom two wealth quintiles) households.

We tested the validity of our PSM method by evaluating matching quality in two ways. First, we examined the difference in mean and median percentage bias across all matching variables (covariates of the first stage probit regression of PSM) before and after matching. Bias measures the differences in the sample mean (median) of a covariate between matched and unmatched groups, calculated as the percentage of the square root of the average (median) of the sample variance of the groups. A reduction in bias indicates the matching procedure has made the two groups more comparable. Second, we examined the pseudo *R*^2^ of the PSM model. The subsample of only matched observations from both groups is taken, then first-stage PSM is conducted again from this subsample providing a pseudo *R*^2^ value. A higher *p* value or lower pseudo *R*^2^ after matching would indicate there is a reduction in systematic differences in variables. All analyses were conducted using Stata version 14.2 and *p* < 0.05 was used for statistical significance.

### Reporting summary

Further information on research design is available in the Nature Portfolio Reporting Summary linked to this article.

## Results

### Summary statistics

Table 1 presents the differences between key demographic and socioeconomic variables between children surveyed before and after the COVID-19 lockdown. There were 73,349 post-COVID and 159,571 pre-COVID under-5 children in NFHS-5. Across the country, there were more post-COVID children in the Central region (51% vs. 14%, *p* < 0.01) and fewer in the Northeast region (4% vs. 19%, *p* < 0.01), relative to pre-COVID groups. Other variables with large significant differences include the greater number of post-COVID children in Hindu households (81% vs. 70%, *p* < 0.01) and with mothers completing higher education (16% vs. 13%, *p* < 0.01). Children in the post-COVID group were 0.46 months younger (*p* < 0.01) than pre-COVID children.

**Table 1:** Differences in key background characteristics between post-COVID and pre-COVID children

	Post-COVID		Pre-COVID			
	Mean	Standard Deviation	Mean	Standard Deviation	Difference	*P* value
*Region*						
North	0.21	0.40	0.18	0.38	0.03**	0.00
Central	0.51	0.50	0.14	0.35	0.37**	0.00
East	0.17	0.38	0.20	0.40	−0.03**	0.00
Northeast	0.04	0.21	0.19	0.40	−0.15**	0.00
South	0.07	0.25	0.15	0.36	−0.09**	0.00
Rural	0.80	0.40	0.80	0.40	0.00	0.11
*Wealth quintile*						
1 (poorest)	0.29	0.45	0.26	0.44	0.03**	0.00
2	0.21	0.41	0.24	0.43	−0.03**	0.00
3	0.17	0.38	0.20	0.40	−0.03**	0.00
4	0.16	0.37	0.17	0.38	−0.01**	0.00
5 (richest)	0.16	0.37	0.12	0.32	0.04**	0.00
*Religion*						
Hindu	0.81	0.39	0.70	0.46	0.11**	0.00
Muslim	0.11	0.31	0.16	0.37	−0.05**	0.00
Sikh	0.03	0.18	0.10	0.30	−0.07**	0.00
Christian	0.02	0.15	0.01	0.12	0.01**	0.00
Other	0.03	0.16	0.02	0.15	0**	0.00
*Caste*						
SC	0.22	0.41	0.20	0.40	0.02**	0.00
ST	0.21	0.41	0.20	0.40	0.01**	0.00
OBC	0.41	0.49	0.37	0.48	0.04**	0.00
Other	0.16	0.37	0.23	0.42	−0.07**	0.00
More than 4 household members	0.76	0.43	0.73	0.44	0.02**	0.00
Female head	0.14	0.35	0.16	0.36	−0.02**	0.00
Married	0.99	0.11	0.98	0.13	0**	0.00
*Mother’s education*						
Primary	0.12	0.33	0.13	0.34	−0.01**	0.00
Secondary	0.49	0.50	0.53	0.50	−0.03**	0.00
Higher	0.16	0.36	0.13	0.33	0.03**	0.00
*Household head’s education*						
Primary	0.19	0.39	0.19	0.39	−0.01**	0.00
Secondary	0.42	0.49	0.42	0.49	0.00	0.24
Higher	0.08	0.27	0.08	0.27	0**	0.01
Mother’s height	1.52	0.06	1.52	0.06	0*	0.03
Mother’s age	27.35	4.83	27.30	5.13	0.04+	0.07
Household head’s age	45.63	15.08	45.44	15.28	0.19**	0.00
Female child	0.48	0.50	0.48	0.50	0+	0.09
Child age (months)	29.43	17.51	29.89	17.44	−0.46**	0.00
Child’s birth order						
1	0.38	0.49	0.38	0.49	0.00	0.10
2	0.33	0.47	0.33	0.47	0.00	0.36
3	0.16	0.37	0.15	0.36	0.01**	0.00
>3	0.13	0.34	0.13	0.34	0.00	0.91
Institutional birth	0.88	0.32	0.85	0.35	0.03**	0.00
Sample size	73,349		159,571			

Data are from the National Family Health Survey 2019–2021 (NFHS-5). Children under the age of five years were included. Children who were surveyed after March 25, 2020 (the first day of national COVID-19 lockdown) were considered post-COVID while those surveyed earlier were considered pre-COVID. +*p* < 0.1, *p < 0.05, **p < 0.01.

### Estimates of the associations of the pandemic with child growth outcomes

Table 2 presents the propensity score matching (PSM)-based (one-to-one nearest neighbor matching) summary estimates of the associations of the pandemic with child growth outcomes. Estimates are reported separately for all post-COVID children (surveyed in 2020 and 2021) and post-COVID children surveyed in 2020. The same sample of pre-COVID children was used as the comparison group for both analyses.

**Table 2:** Propensity score matching-based estimates of the effect of the COVID-19 pandemic on child growth outcomes in India

Exposure	Outcome	ATT estimator of the associations of the COVID-19 pandemic	*P* value	Lower bound	Upper Bound	Sample size
2020 and 2021	Stunting (%)	−0.6	0.16	−1.4	0.2	109,947
	Wasting (%)	−1.2	0.00	−1.9	−0.5	107,907
	Underweight (%)	1.2	0.00	0.4	1.9	112,066
	Height for age (z-score)	−0.10	0.00	−0.13	−0.06	109,933
	Weight for height (z-score)	−0.04	0.00	−0.07	−0.01	107,907
2020	Stunting (%)	1.6	0.02	0.2	2.9	59,466
	Wasting (%)	2.4	0.00	1.3	3.5	58,005
	Underweight (%)	4.6	0.00	3.4	5.9	60,856
	Height for age (z-score)	−0.07	0.01	−0.12	−0.01	59,457
	Weight for height (z-score)	−0.03	0.17	−0.08	0.01	58,005

*HAZ* height-for-age z-score, *WHZ* weight-for-height z-score.

Data are from the National Family Health Survey 2019–2021 (NFHS-5). Children under the age of five years were included. Children who were surveyed after March 25, 2020 (the first day of national COVID-19 lockdown) were considered post-COVID while those surveyed earlier were considered pre-COVID. The estimated effect of the pandemic is the average treatment effect on the treated (ATT) estimator of propensity score matching (one-to-one nearest neighbor matching with replacement).

During 2020 and 2021, post-COVID children had 1.2% (95% CI: 0.5–1.9%, *p* < 0.01) higher underweight rates, 0.10% (95% CI: 0.06–0.13, *p* < 0.01) lower height-for-age Z-scores, and 0.04 (95% CI: 0.01–0.07, *p* < 0.01) lower weight-for-height Z-scores as compared with matched pre-COVID children. However, wasting rates were 1.2% (95% CI: 0.5%–1.9%, *p* < 0.01) lower in post-COVID children as compared with the matched comparison group.

During 2020, post-COVID children had 4.6% (95% CI: 3.4%–5.9%, *p* < 0.01), 1.6% (95% CI: 0.2%–2.9%, *p* < 0.05), and 2.4% (95% CI: 1.3%–3.5%, *p* < 0.01) higher underweight, stunting, and wasting rates, respectively than matched pre-COVID children. They also had 0.07 (95% CI: 0.01–0.12, *p* < 0.05) lower height-for-age Z-scores than matched pre-COVID children.

These estimates were similar in sensitivity analyses in which we used two alternative matching algorithms—three nearest three neighbor matching and kernel matching—instead of one-to-one nearest neighbor matching (Supplementary Tables 1 and 2). The results were also not sensitive to the inclusion of past state-level nutrition trends (anthropometric indicators from NFHS 2015-2016 included as covariates), as presented in Table 3.

**Table 3:** Propensity score matching-based estimates of the effect of the COVID-19 pandemic on child growth outcomes in India, employing NFHS-4 controls

Exposure	Outcome	ATT estimator of the associations of the COVID-19 pandemic	*P* value	Lower bound	Upper Bound	Sample size
2020 and 2021	Stunting (%)	−0.6	0.16	−1.4	0.2	109,947
	Wasting (%)	−1.2	0.00	−1.9	−0.5	107,907
	Underweight (%)	1.2	0.00	0.4	1.9	112,066
	Height for age (z-score)	−0.10	0.00	−0.13	−0.06	109,933
	Weight for height (z-score)	−0.04	0.00	−0.07	−0.01	107,907
2020	Stunting (%)	1.6	0.02	0.2	2.9	59,466
	Wasting (%)	2.4	0.00	1.3	3.5	58,005
	Underweight (%)	4.6	0.00	3.4	5.9	60,856
	Height for age (z-score)	−0.07	0.01	−0.12	−0.01	59,457
	Weight for height (z-score)	−0.03	0.17	−0.08	0.01	58,005

*HAZ* height-for-age z-score, *WHZ* weight-for-height z-score.

Data are from the National Family Health Survey 2019–2021 (NFHS-5). Children under the age of five years were included. Children who were surveyed after March 25, 2020 (the first day of national COVID-19 lockdown) were considered post-COVID while those surveyed earlier were considered pre-COVID. The estimated effect of the pandemic is the average treatment effect on the treated (ATT) estimator of propensity score matching (one-to-one nearest neighbor matching with replacement). In this alternative model, we included state level stunting, wasting, and underweight rates from NFHS-4 data as covariates.

### Subsample analysis

Table 4 presents the subsample results by wealth group, rural or urban location, and sex of the child. In our analysis with children surveyed in both 2020 and 2021, post-COVID children in the rural and low-wealth (two poorest wealth quintile) subsamples were more likely to be underweight than matched pre-COVID children, while no differences in underweight rates were seen in high-wealth and urban subsamples. Height-for-age z-scores were lower among post-COVID children across all subsamples, and the largest differences with pre-COVID children were in low-wealth households (−0.17) followed by rural households (−0.06).

**Table 4:** Propensity score results of effect of COVID-19 pandemic on child health outcomes, subsample analysis

Exposure		2020 and 2021					2020				
Sample	Outcome	ATT estimator of the associations of the COVID-19 pandemic	*P* value	Lower bound	Upper Bound	Sample size	ATT estimator of the associations of the COVID-19 pandemic	*P* value	Lower bound	Upper Bound	Sample size
High-income	Stunting (%)	−0.5	0.32	−1.6	0.5	56,459	3.0	0.00	1.2	4.7	31,320
	Wasting (%)	−1.7	0.00	−2.6	−0.8	55,392	0.4	0.55	−1.0	1.9	30,620
	Underweight (%)	0.8	0.12	−0.2	1.8	57,481	4.4	0.00	2.8	6.1	32,001
	Height for age (z-score)	−0.06	0.01	−0.10	−0.01	56,457	−0.05	0.15	−0.12	0.02	31,318
	Weight for height (z-score)	0.02	0.41	−0.02	0.05	55,392	0.00	0.91	−0.06	0.06	30,620
Low-income	Stunting (%)	0.7	0.25	−0.5	2.0	53,488	1.9	0.06	−0.1	3.9	28,146
	Wasting (%)	−1.2	0.02	−2.2	−0.2	52,515	4.2	0.00	2.5	5.9	27,385
	Underweight (%)	2.0	0.00	0.8	3.1	54,585	4.9	0.00	3.0	6.9	28,855
	Height for age (z-score)	−0.17	0.00	−0.21	−0.12	53,476	−0.12	0.00	−0.20	−0.05	28,139
	Weight for height (z-score)	−0.10	0.00	−0.14	−0.06	52,515	−0.14	0.00	−0.20	−0.07	27,385
Urban	Stunting (%)	−0.1	0.91	−1.8	1.6	22,321	2.9	0.04	0.1	5.7	12,444
	Wasting (%)	−2.4	0.00	−3.9	−0.8	21,873	1.5	0.20	−0.8	3.9	12,155
	Underweight (%)	−0.2	0.80	−1.8	1.4	22,779	5.0	0.00	2.5	7.5	12,747
	Height for age (z-score)	−0.08	0.02	−0.15	−0.01	22,321	−0.07	0.21	−0.18	0.04	12,444
	Weight for height (z-score)	0.02	0.46	−0.04	0.09	21,873	0.03	0.50	−0.06	0.13	12,155
Rural	Stunting (%)	−0.3	0.54	−1.2	0.6	87,498	2.1	0.01	0.6	3.6	46,894
	Wasting (%)	−0.9	0.02	−1.7	−0.1	85,906	2.4	0.00	1.1	3.6	45,722
	Underweight (%)	1.7	0.00	0.8	2.6	89,157	5.0	0.00	3.5	6.4	47,979
	Height for age (z-score)	−0.09	0.00	−0.13	−0.06	87,484	−0.11	0.00	−0.17	−0.05	46,885
	Weight for height (z-score)	−0.07	0.00	−0.10	−0.04	85,906	−0.06	0.03	−0.11	0.00	45,722
Male	Stunting (%)	0.5	0.39	−0.6	1.6	57,097	3.0	0.00	1.1	4.9	30,825
	Wasting (%)	−0.5	0.33	−1.4	0.5	55,936	2.5	0.00	0.9	4.0	29,981
	Underweight (%)	1.6	0.00	0.5	2.7	58,253	5.5	0.00	3.7	7.3	31,592
	Height for age (z-score)	−0.12	0.00	−0.16	−0.07	57,089	−0.09	0.01	−0.16	−0.02	30,819
	Weight for height (z-score)	−0.07	0.00	−0.11	−0.04	55,936	−0.03	0.37	−0.09	0.03	29,981
Female	Stunting (%)	−1.0	0.11	−2.1	0.2	52,850	−0.6	0.53	−2.5	1.3	28,641
	Wasting (%)	−1.1	0.02	−2.0	−0.2	51,971	2.5	0.00	1.0	4.0	28,024
	Underweight (%)	0.9	0.13	−0.2	2.0	53,813	4.1	0.00	2.3	5.9	29,264
	Height for age (z-score)	−0.11	0.00	−0.15	−0.06	52,844	−0.06	0.10	−0.14	0.01	28,638
	Weight for height (z-score)	−0.06	0.00	−0.09	−0.02	51,971	−0.05	0.15	−0.11	0.02	28,024

*HAZ* height-for-age z-score, *WHZ* weight-for-height z-score.

Data are from the National Family Health Survey 2019-2021 (NFHS-5). Children under the age of five years were included. Children who were surveyed after March 25, 2020 (the first day of national COVID-19 lockdown) were considered post-COVID while those surveyed earlier were considered pre-COVID. The estimated effect of the pandemic is the average treatment effect on the treated (ATT) estimator of propensity score matching (one-to-one nearest neighbor matching with replacement). Low-wealth households belonged to the two poorest wealth quintiles, while high-wealth households belonged to the three richest wealth quintiles.

When we separately considered children surveyed in 2020, rural and low-wealth post-COVID children were more likely to be wasted as compared to matched pre-COVID children from the corresponding subgroups, while wasting rates in urban and high-wealth post-COVID children were not different from their matched pre-COVID counterparts. Stunting rates were higher in post-COVID children than matched pre-COVID children across all subsamples except for low-wealth households and girls, where the difference was not statistically significant. In all subsamples, underweight rates were higher in post-COVID children than in the matched comparison group.

Differences in growth indicators between post-COVID and matched pre-COVID groups were larger for boys as compared with girls. Our subsample estimates were not sensitive to alternative three nearest neighbors and kernel matching algorithms (Supplementary Tables 3 and 4).

### Matching quality test results

PSM substantially reduced systematic differences between the post-COVID and pre-COVID groups. There were substantial reductions in mean and median percentage bias in the values of the covariates from the unmatched data to the matched sample. The goodness of fit of the propensity score estimation model (pseudo *R*^2^) was substantially lower in the matched sample than the unmatched data — the pseudo R^2^ values reduced from 0.08–0.10 in all models to 0.00 (Supplementary Tables 5 and 6). The results show that our PSM estimator was valid^39–41^.

## Discussion

The COVID-19 pandemic has caused substantial disruptions in food, health, and economic systems globally, causing reductions in health service utilization and access to nutritious foods. We used national health survey data in India to estimate the potential effect of the pandemic on child health and nutritional status. We found that after accounting for socioeconomic factors, weight and height indicators of under-5 children surveyed after the pandemic were worse as compared with children surveyed before the pandemic. The largest differences were concentrated in children from rural and low-income households. The differences between the two groups were also larger when only children surveyed in 2020 were considered, suggesting a partial recovery in nutritional status of children in 2021.

There is limited empirical evidence with which we could compare our results. Our findings are consistent with one yet unpublished study^25^ in the Indian context that estimated that children born during the pandemic had significantly lower birth weight than those born before the pandemic. Another study from a single health center in Mumbai found that preterm birth rates decreased from 14% to 10% from the first wave to the second wave of the pandemic, although the authors did not account for confounding factors^26^. In the South Asia region, a study from a tertiary health center in Dhaka, Bangladesh, examined 9290 hospitalized children and found higher stunting, wasting, and risk of mortality in children under six months of age who were admitted to the hospital during the COVID-19 pandemic compared to children admitted pre-pandemic^42^.

Globally, several studies have focused on preterm birth rates and neonatal birth weight during the pandemic. A 2022 meta-analysis of 66 studies—more than 70% of which were from upper middle- or high-income countries including four from China—found that there was a significant reduction in preterm birth rates during the pandemic^43^. Most studies included in both meta-analyses used single health center data. Besides birth outcomes, studies focusing on children have primarily analyzed weight changes in school age children (older than five years of age) and adolescents in higher-income countries^44–46^. A 2023 meta-analysis^43^ of 36 studies estimated that mean birth weight increased during the COVID-19 pandemic, but there was no change among children in LMICs. There were 15 LMIC studies included in this meta-analysis, with six studies from China. Most studies included in the meta-analysis used single health center data, unlike ecological or population-level data used in our analysis.

Previous modeling studies^13,15,23,24^ conducted in the early stages of the pandemic had predicted negative impacts of the pandemic on nutritional status. One study estimated that COVID-19 may cause an additional 9.3 million wasted children, 2.6 million stunted children, and 168,000 additional child deaths by 2022 globally due to disruptions in health and economic systems^15^. Future productivity losses of $29.7 billion were estimated globally due to increased stunting and mortality^15^. Another projection based on a model of 118 LMICs predicted at least 253,500 additional child deaths over a six month period, of which 18–23% would be due to increased child wasting and 41% due to reduced access to antibiotics for pneumonia and neonatal sepsis and oral rehydration solution for diarrhea^13^. An analysis of economic disruptions in 129 countries estimated that a 5% reduction in gross domestic product per capita in 2020 would have caused 282,996 additional deaths in under-5 children^24^. With 43,063 under-5 deaths, India was the largest contributor to this estimated burden, and the deaths were estimated to double for every additional 5% decrease in economic activity^24^.

Our estimates suggest that the worst-case scenarios predicted by modeling studies were not realized in India in the short-term. We found an increase in probability of stunting of 1.6% by the end of 2020, but a partial recovery in 2021. Height-for-age z-scores did not fully recover during this study period, with a reduction of 0.1 through mid-2021. Underweight rate among pandemic-affected children was 1.2% higher, with weight-for-height z-scores decreasing by 0.04 at the end of the study period. These estimates are equivalent to an additional 1.4. million underweight under-5 children in 2021 or a loss of two years of progress in underweight rate reduction based on historical progress. Both wasting rates and WHZ were lower in the post-pandemic period up to April 2021, relative to the pre-pandemic periods. Therefore, the distribution of weight-for-height improved for those with the lowest WHZ, but decreased everywhere else. In sub-sample analysis, rise in child underweight rates was observed only in rural and low-wealth households. Children in low-wealth households experienced the highest reduction in height-for-age and weight-for-height z-scores.

Our study has important policy implications. According to the fetal origins hypothesis, shocks to child health—such as acute or sustained malnutrition—during early development stages can have long-lasting effects^21,22^. Stunting and wasting have been associated with fewer years of schooling completed, poor cognition, greater risk of mortality, and lower wages^47^. Wasting and stunting in children is associated with a 12-fold increased mortality risk^19^. Increases in stunting may have resulted in a 7% reduction in optimal cognitive function in Africa and South Asia. Childhood stunting is estimated to cause annual losses of 9–10% GDP per capita when stunted children reach adulthood^48^. Catch-up growth is possible with proper nutrition after initial stunting, but is unable to completely undo the total damage to child development and developmental epigenetics due to shocks during sensitive growth periods.

The COVID-19 pandemic may have adversely affected child nutrition indicators through several mechanisms: 1) disruptions to food systems and the food supply chain may have limited the availability of foods for mothers during pregnancy and in postpartum, and for their children, 2) access to maternal and child health services may have been limited during the pandemic, especially due to lockdowns, and 3) mothers may have been infected by COVID-19 during pregnancy or in the postpartum period, affecting child health^49,50^. As there are a myriad of factors potentially affecting child nutrition, a multipronged approach will be necessary to recover from the damage caused to child health by the pandemic.

First, in Asia and Africa, where 80% of food consumption relies on the supply chain^51^, investments to enhance food and health system resilience are crucial to mitigate shocks such as the COVID-19 pandemic^16^. The pandemic led to reduced food supply due to production restrictions and hoarding. By May 2020, wheat and rice prices in India increased by 4% and 11%^51^, and prices of grocery staples like potatoes and tomatoes increased by 15% and 28%, respectively, from the pre- to post-lockdown period^52^. Longer global food supply chains were more susceptible than shorter ones^53^. In Andhra Pradesh, household food insecurity rose from 21% in December 2019 to 80% in August 2020^54^. Children in food-insecure households were almost half as likely to have a diverse diet (at least four of the seven food groups consumed in a 24 h period) as compared with food-secure households^54^. Despite expanded government initiatives such as free rations, only half of households received food supplementation, with consistently food-insecure households having lower access^54^. A case study on Maharashtra found that the closure of wholesale markets disrupted supply chains and producers faced challenges due to financial and resource constraints, resulting in higher food prices^55^. A robust local agricultural production and delivery system, including a strengthened public food distribution system, is essential to prevent similar food availability shocks.

Second, access to maternal and child health and nutrition programs must be fully restored to their pre-pandemic levels. During the pandemic, programs that directly provide food to mothers and children were affected. Nationwide, schools were closed for over 18 months and the daily free school lunch program (known as the mid-day meal scheme)—which is a major source of supplementary child nutrition in India—was suspended^56,57^. Additionally, some mothers and their children may have delayed healthcare seeking during the pandemic, resulting in reductions in antenatal care visits and institutional childbirths^11,58^. Maternal malnutrition during pregnancy and post-pregnancy, poor feeding and care practices, and childhood infections may be associated with lower maternal care access, which in turn may negatively affect child nutritional status^59^.

Finally, universalizing key childhood health programs such as routine immunization is critical. Previous work has shown positive relationships between routine childhood vaccinations and anthropometric outcomes of children in India^60–62^. Despite the role vaccines play in improving child growth outcomes, the coverage of DPT3 (diphtheria, pertussis, and tetanus, third dose) vaccine among Indian children reduced from 91% in 2019 to 85% in 2020^63^. Globally, an estimated 23 million children did not receive DPT3 in 2020^63^. There is an urgent need for catch-up vaccinations for missed doses and continued efforts towards universal coverage.

These efforts will require a multisectoral approach and international support^15^. However, funding to multilateral organizations such as the WHO, UNICEF, and the World Food Programme, may decrease during a crisis. Increased pressure on donor countries and the ability to mobilize domestic resources will be key. Increased investments for these interventions is required immediately—a study suggested an additional $1.2 billion per year will be needed to meet global nutrition targets due to the COVID-19 pandemic, on top of the previously $7 billion estimated need^15^.

There are important limitations to our analysis. While we accounted for a wide range of potentially confounding factors in our propensity score matching, there may remain unobserved characteristics of children that are different between the pre-COVID and post-COVID groups. If such differences are correlated with child growth outcomes, they may bias our estimates. Second, while we have primarily focused on undernutrition in the context of weight and height indicators, it is also possible that some children may have experienced increased rates of overweight and obesity due to reduced physical activity during the lockdown. A meta-analysis of 15 countries found a link between COVID-19 lockdowns and rates of obesity and overweight among children and adolescents^46^. Third, because the NFHS-5 survey ended in April 2021, out study could not capture the potential negative effects of the pandemic during the COVID-19 delta variant surge and related lockdowns and other measures during April to June of 2021 in India. Fourth, our work could not identify the mechanisms through which the identified changes occurred. For example, we could not measure the relative contribution of environmental stressors, infections, and lack of nutrition in reducing a child’s nutritional status. Finally, we considered height and weight outcomes as they are the most commonly used and reported growth indicators. Other biomarkers such as head circumference and anemia rates could also be examined in the future.

The COVID-19 pandemic led to decreases in anthropometric outcomes of children during their sensitive developmental stages. A partial recovery in child health outcomes was observed in 2021; child height and weight have not fully recovered, and the effects are concentrated in vulnerable households. The resilience of health and food systems to shocks such as COVID-19 should be strengthened while immediate investments are required to decrease child malnutrition and improve broader child health outcomes.

### Supplementary information

Supplementary Information

Reporting Summary

## Data Availability

Raw household survey data are publicly available from the Demographic and Health Surveys, https://dhsprogram.com/data/. Source data for this study are available from Dataverse^64^.

## Code availability

Code is available from Dataverse^64^.
