# Supplementary material for: Analysis of anthropometric outcomes in Indian children during the COVID-19 pandemic using National Family Health Survey data
Source: Commun Med (Lond). 2024 Jul 1;4:127. doi: 10.1038/s43856-024-00543-6 (PMC11217468; doi:10.1038/s43856-024-00543-6)
Supplement: Supplementary file 1 — Supplementary Information [file 43856_2024_543_MOESM1_ESM.pdf]

Supplementary Tables for:

Analysis of anthropometric outcomes in Indian children during the COVID-19 pandemic using National Family Health Survey data

Amit Summan[1], Arindam Nandi[1 2], Ramanan Laxminarayan [3 4]

1. One Health Trust, 5636 Connecticut Avenue NW, PO Box 42735, Washington, DC, 20015, USA
2. The Population Council, 1 Dag Hammarskjold Plaza. New York, NY 10017, USA; Email: anandi@popcouncil.org
3. One Health Trust, Obeya Pulse, First Floor, 7/1, Halasur Road Bengaluru, Karnataka 560042, India
4. High Meadows Environmental Institute, Princeton University, Guyot Hall, Princeton, NJ 08544, USA

Supplementary Table 1: Propensity score matching estimates of the effect of the COVID-19 pandemic on child growth outcomes in India, matching to nearest 3 neighbors.

Pandemic time period	Outcome	Estimated effect of the pandemic	P-value	95% confidence interval	Sample size
2020 and 2021	Stunting	-0.6	0.10	-1.3, 0.1	109,947
	Wasting	-1.2	0.00	-1.8, -0.6	107,907
	Underweight	1.3	0.00	0.6, 1.9	112,066
	HAZ	-0.092	0.00	-0.119, -0.065	109,933
	WHZ	-0.043	0.00	-0.065, -0.02	107,907
2020	Stunting	1.5	0.01	0.4, 2.6	59,466
	Wasting	2.2	0.00	1.3, 3.2	58,005
	Underweight	4.8	0.00	3.7, 5.9	60,856
	HAZ	-0.071	0.00	-0.114, -0.027	59,457
	WHZ	-0.034	0.07	-0.071, 0.003	58,005

Note: *HAZ*: height-for-age z-score; *WHZ*: weight-for-height z-score.
Data are from the National Family Health Survey 2019-2021 (NFHS-5). Children under the age of five years were included. Children who were surveyed after March 25, 2020 (the first day of national COVID-19 lockdown) were considered post-COVID while those surveyed earlier were considered pre-COVID. The estimated effect of the pandemic is the average treatment effect on the treated (ATT) estimator of propensity score matching (3 nearest neighbors with replacement).

Supplementary Table 2: Propensity score matching estimates of the effect of the COVID-19 pandemic on child growth outcomes in India, kernel matching.

Pandemic time period	Outcome	Estimated effect of the pandemic	P-value	95% confidence interval	Sample size
2020 and 2021	Stunting	-0.6	0.10	-1.3, 0.1	109,947
	Wasting	-1.2	0.00	-1.8, -0.6	107,907
	Underweight	1.3	0.00	0.6, 1.9	112,066
	HAZ	-0.092	0.00	-0.119, -0.065	109,933
	WHZ	-0.043	0.00	-0.065, -0.02	107,907
2020	Stunting	1.5	0.01	0.4, 2.6	59,466
	Wasting	2.2	0.00	1.3, 3.2	58,005
	Underweight	4.8	0.00	3.7, 5.9	60,856
	HAZ	-0.071	0.00	-0.114, -0.027	59,457
	WHZ	-0.034	0.07	-0.071, 0.003	58,005

Note: *HAZ*: height-for-age z-score; *WHZ*: weight-for-height z-score.
Data are from the National Family Health Survey 2019-2021 (NFHS-5). Children under the age of five years were included. Children who were surveyed after March 25, 2020 (the first day of national COVID-19 lockdown) were considered post-COVID while those surveyed earlier were considered pre-COVID. The estimated effect of the pandemic is the average treatment effect on the treated (ATT) estimator of propensity score matching (kernel matching).

Supplementary Table 3: Propensity score matching estimates of the effect of the COVID-19 on child growth outcomes, subsample analysis with matching to 3 nearest neighbors.

		2020 and 2021 Pandemic period				2020 Pandemic period			
Sample	Outcome	Estimated effect of the pandemic	P-value	95% confidence interval	Sample size	Estimated effect of the pandemic	P-value	95% confidence interval	Sample size
High-wealth	Stunting	-0.8	0.09	-1.7, 0.1	56,459	2.4	0.00	0.9, 3.8	31,320
	Wasting	-1.8	0.00	-2.6, -1.1	55,392	1.0	0.12	-0.2, 2.2	30,620
	Underweight	0.6	0.18	-0.3, 1.4	57,481	4.0	0.00	2.6, 5.4	32,001
	HAZ	-0.08	0.00	-0.12, -0.05	56,457	-0.07	0.01	-0.13, -0.02	31,318
	WHZ	0.02	0.28	-0.01, 0.05	55,392	-0.01	0.84	-0.06, 0.05	30,620
Low-wealth	Stunting	0.3	0.54	-0.7, 1.4	53,488	1.8	0.04	0.1, 3.4	28,146
	Wasting	-0.9	0.03	-1.8, -0.1	52,515	2.9	0.00	1.5, 4.3	27,385
	Underweight	2.3	0.00	1.3, 3.3	54,585	4.9	0.00	3.3, 6.6	28,855
	HAZ	-0.14	0.00	-0.18, -0.1	53,476	-0.11	0.00	-0.17, -0.04	28,139
	WHZ	-0.09	0.00	-0.12, -0.06	52,515	-0.08	0.00	-0.14, -0.03	27,385
Urban	Stunting	0.0	1.00	-1.5, 1.5	22,321	2.7	0.03	0.3, 5.1	12,444
	Wasting	-2.7	0.00	-4, -1.4	21,873	2.1	0.04	0.1, 4	12,155
	Underweight	0.5	0.49	-0.9, 1.9	22,779	3.8	0.00	1.6, 6	16,807
	HAZ	-0.09	0.00	-0.15, -0.03	22,321	-0.06	0.17	-0.16, 0.03	12,444
	WHZ	0.06	0.04	0, 0.11	21,873	0.02	0.69	-0.06, 0.1	12,155
Rural	Stunting	-0.1	0.79	-0.9, 0.7	87,498	1.7	0.01	0.5, 3	46,894
	Wasting	-0.9	0.01	-1.5, -0.3	85,906	2.0	0.00	0.9, 3.1	45,722
	Underweight	1.6	0.00	0.9, 2.4	89,157	4.7	0.00	3.4, 5.9	47,979
	HAZ	-0.10	0.00	-0.13, -0.07	87,484	-0.08	0.00	-0.13, -0.03	46,885
	WHZ	-0.07	0.00	-0.09, -0.04	85,906	-0.04	0.04	-0.09, 0	45,722
Boys	Stunting	0.3	0.59	-0.7, 1.2	57,097	2.6	0.00	1, 4.2	30,825
	Wasting	-1.0	0.01	-1.8, -0.2	55,936	2.4	0.00	1, 3.7	29,981
	Underweight	1.3	0.00	0.4, 2.3	58,253	5.3	0.00	3.8, 6.8	31,592
	HAZ	-0.11	0.00	-0.15, -0.08	57,089	-0.09	0.00	-0.15, -0.03	30,819
	WHZ	-0.05	0.00	-0.09, -0.02	55,936	-0.05	0.05	-0.11, 0	29,981
Girls	Stunting	-1.0	0.04	-2, 0	52,850	0.5	0.56	-1.1, 2.1	28,641
	Wasting	-1.2	0.00	-2, -0.4	51,971	2.4	0.00	1.1, 3.7	28,024
	Underweight	1.1	0.02	0.2, 2.1	53,813	4.0	0.00	2.5, 5.5	29,264
	HAZ	-0.10	0.00	-0.14, -0.06	52,844	-0.08	0.02	-0.14, -0.01	28,638
	WHZ	-0.05	0.00	-1.7, 0.1	51,971	-0.05	0.05	-0.11, 0	28,024

Note: *HAZ*: height-for-age z-score; *WHZ*: weight-for-height z-score.
Data are from the National Family Health Survey 2019-2021 (NFHS-5). Children under the age of five years were included. Children who were surveyed after March 25, 2020 (the first day of national COVID-19 lockdown) were considered post-COVID while those surveyed earlier were considered pre-COVID. The estimated effect of the pandemic is the average treatment effect on the treated (ATT) estimator of propensity score matching (3 nearest neighbors with replacement). Low-wealth households belonged to the two poorest wealth quintiles, while high-wealth households belonged to the three richest wealth quintiles.

Supplementary Table 4: Propensity score matching estimates of the effect of the COVID-19 on child growth outcomes, subsample analysis with kernel matching.

		2020 and 2021 Pandemic period				2020 Pandemic period			
Sample	Outcome	Estimated effect of the pandemic	P-value	Lower bound	Sample size	Average treatment effect	P-value	Lower bound	Sample size
High-wealth	Stunting	-0.8	0.09	-1.7, 0.1	56,459	2.4	0.00	0.9, 3.8	31,320
	Wasting	-1.8	0.00	-2.6, -1.1	55,392	1.0	0.12	-0.2, 2.2	30,620
	Underweight	0.6	0.18	-0.3, 1.4	57,481	4.0	0.00	2.6, 5.4	32,001
	HAZ	-0.08	0.00	-0.12, -0.05	56,457	-0.07	0.01	-0.13, -0.02	31,318
	WHZ	0.02	0.28	-0.01, 0.05	55,392	-0.01	0.84	-0.06, 0.05	30,620
Low-wealth	Stunting	0.3	0.54	-0.7, 1.4	53,488	1.8	0.04	0.1, 3.4	28,146
	Wasting	-0.9	0.03	-1.8, -0.1	52,515	2.9	0.00	1.5, 4.3	27,385
	Underweight	2.3	0.00	1.3, 3.3	54,585	4.9	0.00	3.3, 6.6	28,855
	HAZ	-0.14	0.00	-0.18, -0.1	53,476	-0.11	0.00	-0.17, -0.04	28,139
	WHZ	-0.09	0.00	-0.12, -0.06	52,515	-0.08	0.00	-0.14, -0.03	27,385
Urban	Stunting	0.0	1.00	-1.5, 1.5	22,321	2.7	0.03	0.3, 5.1	12,444
	Wasting	-2.7	0.00	-4, -1.4	21,873	2.1	0.04	0.1, 4	12,155
	Underweight	0.5	0.49	-0.9, 1.9	22,779	3.8	0.00	1.6, 6	16,807
	HAZ	-0.09	0.00	-0.15, -0.03	22,321	-0.06	0.17	-0.16, 0.03	12,444
	WHZ	0.06	0.04	0, 0.11	21,873	0.02	0.69	-0.06, 0.1	12,155
Rural	Stunting	-0.1	0.79	-0.9, 0.7	87,498	1.7	0.01	0.5, 3	46,894
	Wasting	-0.9	0.01	-1.5, -0.3	85,906	2.0	0.00	0.9, 3.1	45,722
	Underweight	1.6	0.00	0.9, 2.4	89,157	4.7	0.00	3.4, 5.9	47,979
	HAZ	-0.10	0.00	-0.13, -0.07	87,484	-0.08	0.00	-0.13, -0.03	46,885
	WHZ	-0.07	0.00	-0.09, -0.04	85,906	-0.04	0.04	-0.09, 0	45,722
Boys	Stunting	0.3	0.59	-0.7, 1.2	57,097	2.6	0.00	1, 4.2	30,825
	Wasting	-1.0	0.01	-1.8, -0.2	55,936	2.4	0.00	1, 3.7	29,981
	Underweight	1.3	0.00	0.4, 2.3	58,253	5.3	0.00	3.8, 6.8	31,592
	HAZ	-0.11	0.00	-0.15, -0.08	57,089	-0.09	0.00	-0.15, -0.03	30,819
	WHZ	-0.05	0.00	-0.09, -0.02	55,936	-0.05	0.05	-0.11, 0	29,981
Girls	Stunting	-1.0	0.04	-2, 0	52,850	0.5	0.56	-1.1, 2.1	28,641
	Wasting	-1.2	0.00	-2, -0.4	51,971	2.4	0.00	1.1, 3.7	28,024
	Underweight	1.1	0.02	0.2, 2.1	53,813	4.0	0.00	2.5, 5.5	29,264
	HAZ	-0.10	0.00	-0.14, -0.06	52,844	-0.08	0.02	-0.14, -0.01	28,638
	WHZ	-0.05	0.00	-0.09, -0.02	51,971	-0.05	0.05	-0.11, 0	28,024

Note: *HAZ*: height-for-age z-score; *WHZ*: weight-for-height z-score.
Data are from the National Family Health Survey 2019-2021 (NFHS-5). Children under the age of five years were included. Children who were surveyed after March 25, 2020 (the first day of national COVID-19 lockdown) were considered post-COVID while those surveyed earlier were considered pre-COVID. The estimated effect of the pandemic is the average treatment effect on the treated (ATT) estimator of propensity score matching (kernel matching). Low-wealth households belonged to the two poorest wealth quintiles, while high-wealth households belonged to the three richest wealth quintiles.

Supplementary Table 5: Covariate balancing between pandemic-affected and matched unaffected children, with one-to-one nearest neighbor propensity score matching.

Pandemic time period	Outcome	Data	Pseudo R^2	Mean bias	Median bias
2020 and 2021	Stunting	Matched	0.00	1.01	0.74
		Unmatched	0.08	5.72	3.09
	Wasting	Matched	0.00	1.08	0.98
		Unmatched	0.08	5.76	3.10
	Underweight	Matched	0.00	0.86	0.63
		Unmatched	0.08	5.69	3.09
	HAZ	Matched	0.00	0.90	0.77
		Unmatched	0.08	5.72	3.09
	WHZ	Matched	0.00	1.08	0.98
		Unmatched	0.08	5.76	3.10
2020	Stunting	Matched	0.00	0.95	0.75
		Unmatched	0.10	6.87	4.98
	Wasting	Matched	0.00	1.02	0.83
		Unmatched	0.09	6.81	4.87
	Underweight	Matched	0.00	0.93	0.82
		Unmatched	0.10	6.87	4.82
	HAZ	Matched	0.00	1.09	0.81
		Unmatched	0.10	6.87	4.99
	WHZ	Matched	0.00	1.02	0.83
		Unmatched	0.09	6.81	4.87

Note: *HAZ*: height-for-age z-score; *WHZ*: weight-for-height z-score.
Data are from the National Family Health Survey 2019-2021 (NFHS-5). Bias measures the differences in the sample mean (median) of a covariate between matched (after PSM) and unmatched (raw data) groups, calculated as the percentage of the square root of the average (median) of the sample variance of the groups.

Supplementary Table 6: Covariate balancing between subsamples of pandemic-affected and matched unaffected children, with one-to-one nearest neighbor propensity score matching.

			2020 and 2021 Pandemic period			2020 Pandemic period		
Sample	Outcome	Data	Pseudo R^2	Mean bias	Median bias	Pseudo R^2	Mean bias	Median bias
High-wealth	Stunting	Matched	0.00	0.88	0.96	0.00	1.25	0.97
		Unmatched	0.10	6.21	3.62	0.11	7.84	5.55
	Wasting	Matched	0.00	1.04	0.77	0.00	1.29	1.11
		Unmatched	0.10	6.19	3.63	0.11	7.77	5.47
	Underweight	Matched	0.00	0.87	0.63	0.00	1.13	0.99
		Unmatched	0.10	6.15	3.44	0.11	7.83	5.44
	HAZ	Matched	0.00	0.98	0.79	0.00	1.02	0.82
		Unmatched	0.10	6.21	3.62	0.11	7.84	5.55
	WHZ	Matched	0.00	1.04	0.77	0.00	1.29	1.11
		Unmatched	0.10	6.19	3.63	0.11	7.77	5.47
Low-wealth	Stunting	Matched	0.00	1.35	1.16	0.00	1.84	1.37
		Unmatched	0.07	7.48	4.91	0.10	8.17	3.96
	Wasting	Matched	0.00	1.40	1.21	0.01	2.15	1.35
		Unmatched	0.07	7.50	4.98	0.10	8.08	3.85
	Underweight	Matched	0.00	1.29	1.06	0.01	2.00	1.64
		Unmatched	0.07	7.40	4.84	0.10	8.12	4.41
	HAZ	Matched	0.00	1.36	1.01	0.01	1.82	0.94
		Unmatched	0.07	7.48	4.91	0.10	8.17	3.94
	WHZ	Matched	0.00	1.40	1.21	0.01	2.15	1.35
		Unmatched	0.07	7.50	4.98	0.10	8.08	3.85
Urban	Stunting	Matched	0.00	1.39	1.26	0.00	1.86	1.56
		Unmatched	0.10	5.89	2.61	0.12	7.10	3.48
	Wasting	Matched	0.00	1.65	1.14	0.00	1.69	1.34
		Unmatched	0.10	5.85	2.53	0.12	7.09	3.65
	Underweight	Matched	0.00	1.37	1.25	0.01	2.37	2.20
		Unmatched	0.10	5.83	2.82	0.19	11.69	7.19
	HAZ	Matched	0.00	1.39	1.26	0.00	1.86	1.56
		Unmatched	0.10	5.89	2.61	0.12	7.10	3.48
	WHZ	Matched	0.00	1.65	1.14	0.00	1.69	1.34
		Unmatched	0.10	5.85	2.53	0.12	7.09	3.65
Rural	Stunting	Matched	0.00	1.10	0.90	0.00	1.13	0.83
		Unmatched	0.08	6.31	3.52	0.10	7.64	5.02
	Wasting	Matched	0.00	1.14	0.91	0.00	1.29	1.15
		Unmatched	0.08	6.33	3.54	0.10	7.56	4.91
	Underweight	Matched	0.00	1.00	0.80	0.00	1.26	1.24
		Unmatched	0.08	6.30	3.54	0.10	7.63	5.09
	HAZ	Matched	0.00	1.17	1.01	0.00	0.84	0.69
		Unmatched	0.08	6.31	3.52	0.10	7.65	5.04
	WHZ	Matched	0.00	1.14	0.91	0.00	1.29	1.15
		Unmatched	0.08	6.33	3.54	0.10	7.56	4.91
Male	Stunting	Matched	0.00	1.15	0.84	0.00	1.38	1.20
		Unmatched	0.08	6.04	3.70	0.10	7.32	5.03
	Wasting	Matched	0.00	1.16	0.96	0.00	1.68	1.61
		Unmatched	0.08	6.07	3.50	0.09	7.26	5.04
	Underweight	Matched	0.00	1.07	0.81	0.00	1.26	1.09
		Unmatched	0.08	6.00	3.87	0.10	7.30	5.36
	HAZ	Matched	0.00	1.29	0.96	0.00	1.26	1.03

		Unmatched	0.08	6.05	3.70	0.10	7.33	5.03
	WHZ	Matched	0.00	1.16	0.96	0.00	1.68	1.61
		Unmatched	0.08	6.07	3.50	0.09	7.26	5.04
Female	Stunting	Matched	0.00	0.90	0.72	0.00	1.22	1.01
		Unmatched	0.08	5.94	3.06	0.10	6.91	4.51
	Wasting	Matched	0.00	0.96	0.71	0.00	1.65	1.38
		Unmatched	0.08	5.94	2.86	0.10	6.80	4.23
	Underweight	Matched	0.00	1.00	0.78	0.00	1.16	1.05
		Unmatched	0.08	5.90	2.76	0.10	6.91	4.87
	HAZ	Matched	0.00	1.02	0.74	0.00	1.35	1.14
		Unmatched	0.08	5.94	3.06	0.10	6.91	4.49
	WHZ	Matched	0.00	0.96	0.71	0.00	1.65	1.38
		Unmatched	0.08	5.94	2.86	0.10	6.80	4.23

Note: *HAZ*: height-for-age z-score; *WHZ*: weight-for-height z-score.
Data are from the National Family Health Survey 2019-2021 (NFHS-5). Bias measures the differences in the sample mean (median) of a covariate between matched (after PSM) and unmatched (raw data) groups, calculated as the percentage of the square root of the average (median) of the sample variance of the groups. Low-wealth households belonged to the two poorest wealth quintiles, while high-wealth households belonged to the three richest wealth quintiles.

Supplementary Table 7: List of states by phase

Phase 1	Phase 2
Andaman & Nicobar Islands	Arunachal Pradesh
Andhra Pradesh	Chandigarh
Assam	Chhattisgarh
Bihar	Delhi
Dadra & Nagar Haveli	Haryana
Goa	Jharkhand
Gujarat	Madhya Pradesh
Himachal Pradesh	Odisha
Jammu & Kashmir	Puducherry
Karnataka	Punjab
Kerala	Rajasthan
Ladakh	Tamil Nadu
Lakshadweep	Uttar Pradesh
Maharashtra	Uttarakhand
Manipur	
Meghalaya	
Mizoram	
Nagaland	
Sikkim	
Telangana	
Tripura	
West Bengal	

Note: All phase 2 states have pre-COVID observations except for Chandigarh.
